# Single-cycle, pseudotyped reporter influenza virus to facilitate evaluation of treatment strategies for avian influenza, Ebola and other highly infectious diseases *in vivo*

**DOI:** 10.3389/fimmu.2025.1608074

**Published:** 2025-07-10

**Authors:** Tiong Kit Tan, Pramila Rijal, Kuan-Ying A. Huang, Stephen C. Hyde, Deborah R. Gill, Alain R. Townsend

**Affiliations:** ^1^ Medical Research Council (MRC) Translational Immune Discovery Unit, Medical Research Council (MRC) Weatherall Institute of Molecular Medicine, University of Oxford, Oxford, United Kingdom; ^2^ Chinese Academy of Medical Science-Oxford Institute, Nuffield Department of Medicine, University of Oxford, Oxford, United Kingdom; ^3^ Graduate Institute of Immunology and Department of Paediatrics, National Taiwan University Hospital, College of Medicine, National Taiwan University, Taipei, Taiwan; ^4^ Gene Medicine Research Group, Nuffield Division of Clinical Laboratory Science, Radcliffe Department of Medicine, University of Oxford, Oxford, United Kingdom

**Keywords:** pandemic, influenza, *in vivo* imaging, bioluminescence, reporter virus

## Abstract

The rapid spread of infectious diseases presents a significant global threat, with seasonal influenza viruses, leading to 290,000–650,000 deaths annually. Emerging high pathogenic influenza strains from animals such as H5N1 and H7N9 further exacerbates pandemic risks. While developing effective vaccines and therapeutics is critical, the evaluation of these interventions is constrained by the requirement for high biosafety containment facilities. To circumvent these challenges, we developed S-Lux, a replication-deficient, single-cycle recombinant influenza virus expressing *firefly luciferase* (*Flux*) as a reporter protein. S-Lux can be pseudotyped with haemagglutinin from avian influenza, H5 and H7, enabling real-time monitoring of viral infection *in vivo*, and facilitate therapeutic antibody evaluation in low-containment facilities. In mice, S-Lux infection resulted in dose-dependent bioluminescent expression in the mouse airways and allowed evaluation of neutralising monoclonal antibodies and clearance of infected cells in mice. To extend this system, we generated ES-Lux by pseudotyping with the Ebola Glycoprotein (GP) and demonstrated that ES-Lux can be used to evaluate the efficacy of Ebola GP-targeting antibodies *in vivo*. Together, S-Lux and ES-Lux enable robust, simple and time-efficient assessment of antiviral therapy targeting influenza and Ebola virus *in vivo*, overcoming biosafety constraints that limit traditional efficacy studies.

## Introduction

Pandemic spread of disease constitutes a serious international threat exacerbated by increased levels of international travel. Influenza viruses, which cause seasonal epidemics leading to 290,000 to 650,000 deaths annually (1), pose a pandemic threat when novel strains arise from antigenic shift or escape from animal reservoirs and transmit among humans. Pandemic influenza outbreaks of high pathogenic avian influenza strains such as H5N1 (2, 3) and H7N9 (4) are thought to pose the greatest risk (5). Much attention has been focused on developing effective vaccines and treatments for influenza and other lethal diseases, but experiments to assess efficacy are restricted to Biosafety Level 3 (BSL-3), or BSL-4 facilities in the case of Ebola virus (EBOV) (5, 6). Limited access to such facilities with its resultant high experimental cost is therefore an obstacle to the discovery and evaluation of new vaccines and treatments for lethal infections.

Conventional animal studies involving influenza viruses are typically restricted to the monitoring of weight loss, clinical signs, survival and viral loads which together often require euthanasia of animals to determine therapeutic efficacy (7, 8). To simplify such experiments, and allow monitoring of real-time infection in animal models, it is convenient to use a bioluminescence reporter system. Several replication-competent, influenza reporter viruses capable of expressing bioluminescence signals upon infection in animal models have been reported (9–14), allowing real-time monitoring of the dynamics of virus spread. Unfortunately, the use of these bioluminescence reporter systems for avian influenza such as H5 or H7 viruses remains restricted to BSL-3 facilities due to their replication-competent nature.

To help address these issues, we have developed a strategy that facilitates the testing of potential treatments to emerging infectious disorders in standard, readily available, BSL-2 facilities. The “S-Lux” system makes use of replication-deficient, single-infectious cycle recombinant influenza viruses, that express *firefly luciferase* (*Flux*) reporter protein upon infection of mammalian cells. The S-Lux system is based on the single-cycle influenza vaccine S-FLU (15), in which the coding sequence for hemagglutinin is replaced with *Flux*. The viral core is coated in a functional hemagglutinin of choice via pseudotyping [reviewed by (16)]. Here, we have generated a prototype S-Lux pseudotyped with H1 hemagglutinin from the commonly used influenza H1N1 A/Puerto Rico/8/1934 Cambridge (PR8) or alternatively pseudotyped with the haemagglutinins from potential pandemic avian influenza strains (H5N1 and H7N9). The infection kinetics of each were characterised *in vivo* including evaluating the sensitivity to known neutralising antibodies. Our data also suggest that the S-Lux system could be a valuable tool for studying influenza virus tropism in the airways, particularly in newly isolated strains or those with pandemic potential. Furthermore, we have expanded the utility of this system by generating “ES-Lux” by pseudotyping with the Ebola Glycoprotein (GP). Our results show that the S-Lux system can serve as a robust, quick, and straightforward approach to investigate the prophylactic efficacy of neutralising antibodies that block influenza and EBOV entry in living animal models without the need for BSL-3 or 4 containment facilities.

## Results

### Generation and characterisation of S-Lux

**Figure 1:**
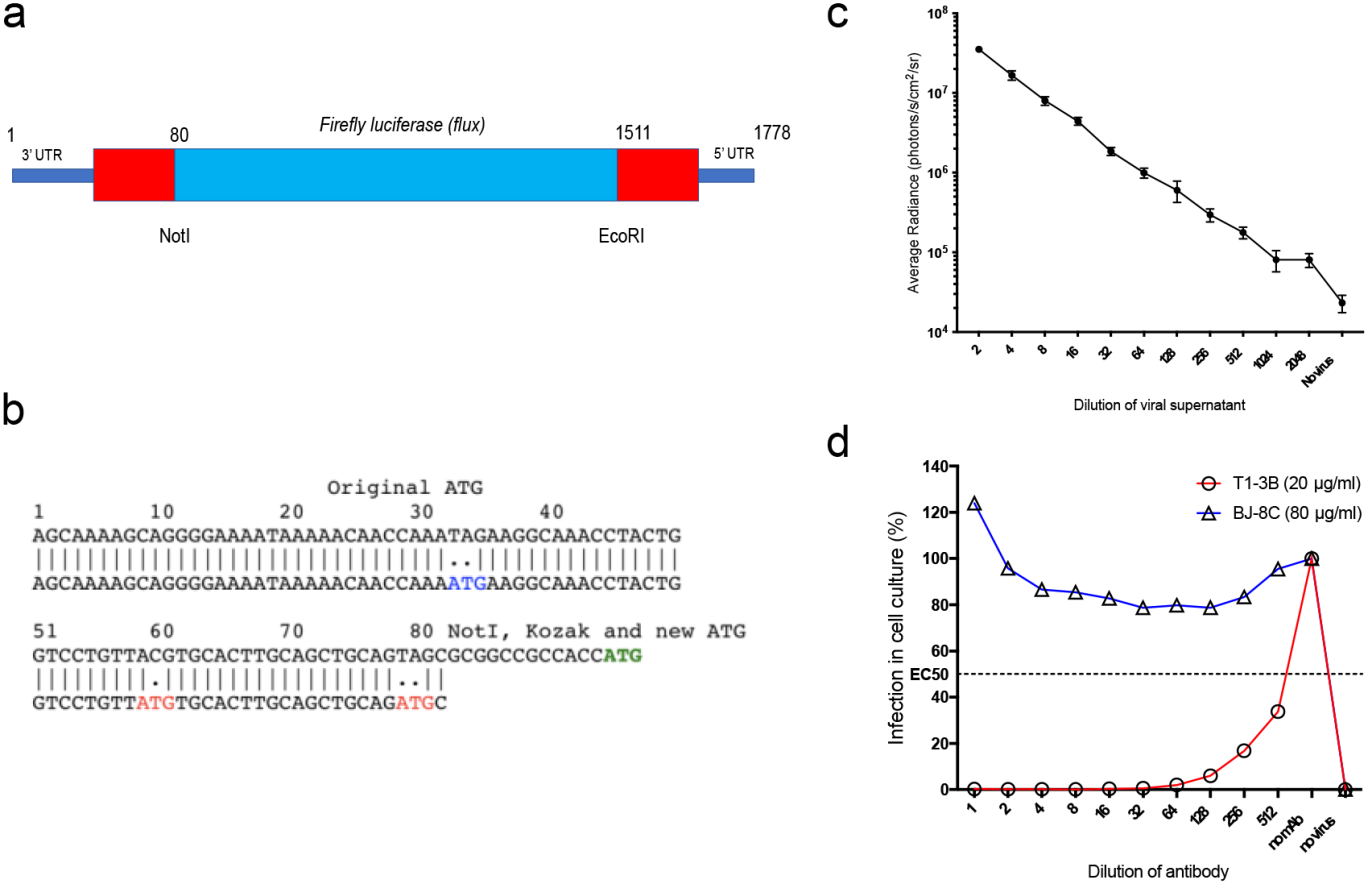
Generation and characterisation of S-Lux *in vitro*. **(a)** Schematic showing the heamagglutinin virus RNA including 5’ and 3’ Untranslated Regions (UTR) flanking the codon-optimised firefly luciferase (*flux*) transgene and the position of *NotI* and *EcoRI* restriction sites. **(b)** The construct was based on S-FLU (15) with modifications to the original 3’ packaging sequence, inactivating two ATG codons (depicted in red) and shortening the 5’ packaging sequence from 1275–1778 to 1511–1778 to allow insertion of a larger transgene. A *NotI* site followed by Kozak sequence were inserted at position 80. The ATG codon (depicted in green) represents the start codon of the inserted *flux* transgene. **(c)** Monolayers of MDCK cells seeded in 96-well plates were infected with dilutions of PR8 S-Lux and bioluminescence was measured 24 h post infection. Each data point represents the average of four readings. **(d)** PR8 S-Lux was incubated with dilutions of a known neutralising antibody (T1-3B), or non-neutralising antibody (BJ-8C), and then added to MDCK cells cultured in 96-well plates. Bioluminescence was measured 24 h post infection. Each data point represents the average of two readings. UTR: untranslated region.

We generated a replication-deficient, single-infectious cycle, recombinant influenza virus, expressing the *firefly luciferase* (*Flux*) reporter protein construct in the well-studied H1N1 PR8 influenza strain background. This was achieved by making specific modifications (**Figures 1a, b**) to the S-HA Flu genome (15) using methods described previously (15, 17). The resulting S-Lux strain retains all PR8 elements except that the hemagglutinin (HA) coding sequence has been replaced with the *flux* coding sequence for firefly luciferase. Viable PR8 S-Lux virus particles were rescued by supplying the A/PR/8/1934 HA gene *in trans* by transient transfection.

The ability of PR8 S-Lux to infect and express *flux* in cultured MDCK cells was confirmed at 24 h with detection of bioluminescence upon addition of D-Luciferin substrate and the signal was inversely proportional to dilutions of PR8 S-Lux (**Figure 1c**). The PR8 S-Lux infection was shown to be blocked by the known H1-neutralising mAb T1-3B (18) in a dose-dependent manner, but was not blocked by a non-relevant mAb (BJ-8C) (**Figure 1d**).

Four alternate S-Lux configurations were subsequently generated based on: (i) the potential pandemic avian influenza H5N1 strain (A/Vietnam/1203/2004), (ii) a 2013 H7N9 isolate [H7 A/Anhui/1/2013 (19)], (iii) a more recent 2017 H7N9 isolate (A/Taiwan/1/2017), and (iv) the 2017 H7N9 isolate (A/Guangdong/TH005/2017). For biosafety reasons, the polybasic HA cleavage site found in each of these four influenza isolates was converted to a monobasic trypsin sensitive site (20) prior to S-Lux pseudotyping. All H5 and H7 S-Lux viruses produced were able to infect cultured MDCK cells and express *flux* in a dose-dependent manner (**Supplementary Figure 1**). Collectively, the generation of these five S-Lux variants supports the notion that inclusion of *flux* transgene in the HA vRNA is well tolerated.

### Bioluminescence expression from S-Lux *in vivo*

**Figure 2:**
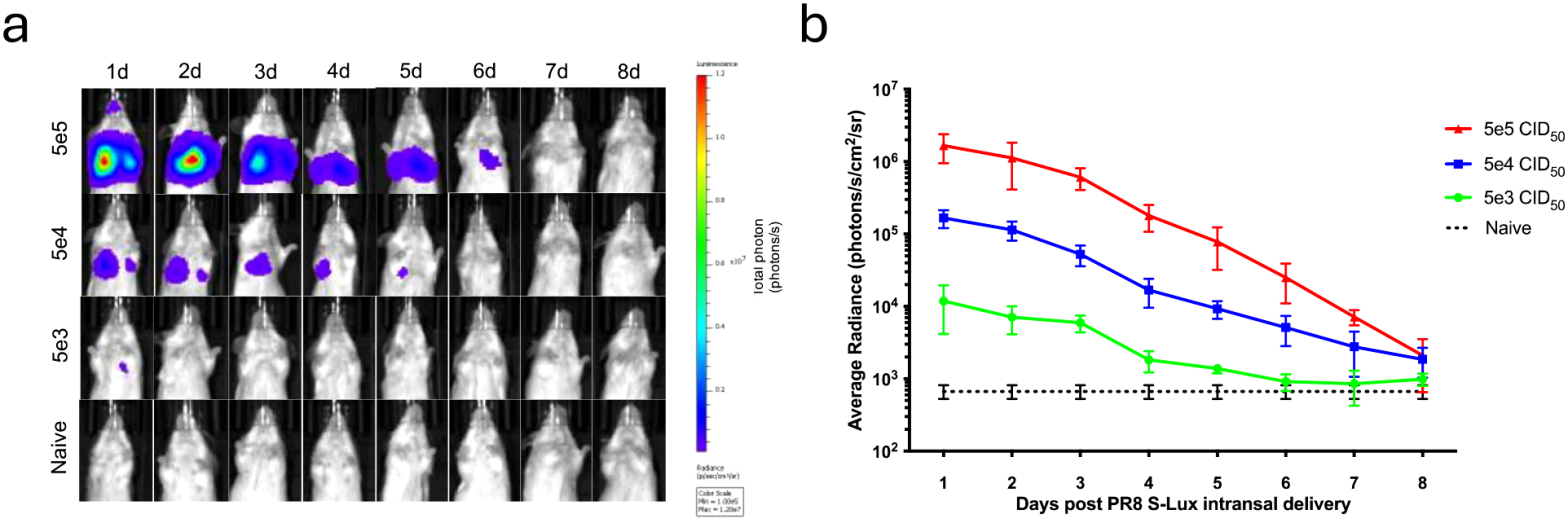
Bioluminescence imaging of mice infected with PR8 S-Lux. The effect of dose response and *flux* kinetics was imaged in mice (n=4) following i.n. infection with 5e3, 5e4 or 5e5 PR8 S-Lux. **(a)** Mice were imaged at the indicated time points; images of only one mouse per group per day is shown. **(b)** Values shown correspond to the average photon flux (photons/s/cm^2^/sr^2^) for each treatment group at indicated time points in **(a)**; each data point represents mean ± s.e.m (n=4).

To evaluate whether S-Lux could lead to bioluminescence which was detectable after *in vivo* infection, mice received an intranasally (i.n) dose of 5e4, 5e5 or 5e6 CID_50_ (median cell infectious dose) of PR8 S-Lux and the level and duration of luciferase bioluminescence in the airways was monitored. At 24 hours post-delivery, bioluminescence was detected in the airways of dosed mice in a dose-dependent fashion (**Figures 2a, b**; p<0.0001; ANOVA with post-test for linear trend). For each of the active dosing groups, bioluminescence peaked at 24 h post-delivery and gradually decreased to background by approximately 8 days post-delivery. Crucially, these results confirm that S-Lux infection can lead to detectable bioluminescence in mice, which in turn can be used as a marker of infection to follow real-time infection kinetics within an animal treatment group. Importantly, the observed gradual decrease of the luciferase signal to background levels confirms that the S-Lux configuration does not support viral replication in the murine lung (15).

### Validation of PR8 S-Lux *in vivo* using a known neutralising antibody

**Figure 3:**
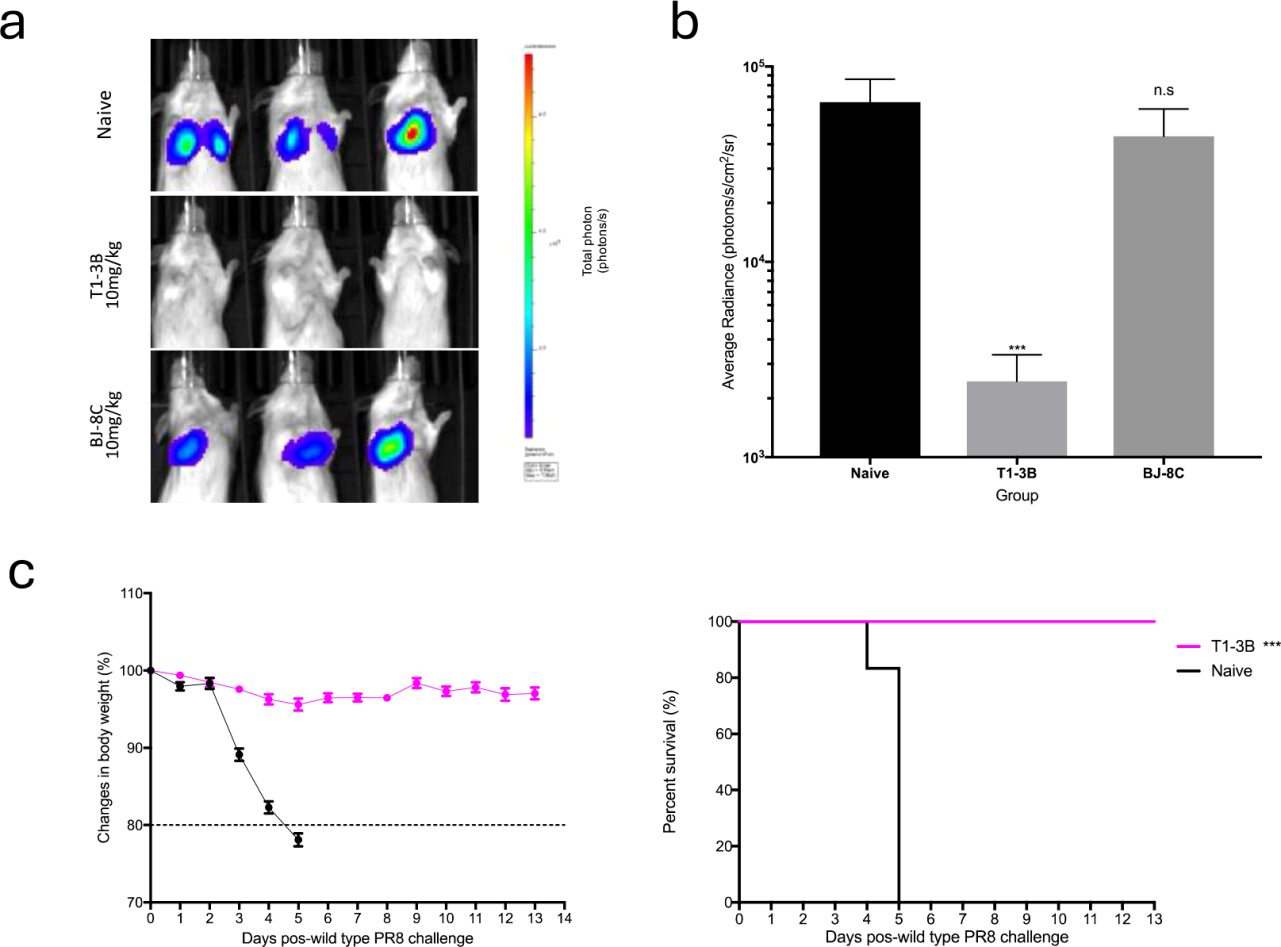
Validation of PR8 S-Lux *in vivo* using known neutralising antibodies. Mice (n=3) were administered i.p. 10 mg/kg of a known neutralising antibody (T1-3B), a non-relevant antibody (BJ-8C), or PBS at 24 h prior to infection. Mice were then dosed i.n. with ~2e4 CID_50_ of PR8 S-Lux and **(a)** imaged at 24 h post infection. **(b)** Values represent the average photon flux (photons/s/cm^2^/sr^2^) as shown in **(a)**; each data point represents mean ± s.e.m (n=3). The statistical significance of differences was calculated using Students’ t-test. **(c)** Mice (n=6) were treated i.p. with 10 mg/kg of T1-3B at 24 h prior to infection or remained naive. Mice were then dosed i.n. with 1e4 TCID_50_ (1,000 LD_50_) of wild type PR8 virus and monitored for weight loss and survival over indicated time point. The statistical significance of differences was calculated using Log-rank test. ***p<0.01, ns, not significant.

As proof of principle for the use of PR8 S-Lux as a surrogate to study anti-influenza treatments, we evaluated the *in vivo* efficacy of well characterised HA neutralising antibodies to limit S-Lux infection. Mice received an intraperitoneal (i.p) dose of 10 mg/kg of T1-3B, a known H1 HA stem neutralising antibody (18) or, as a negative control, a non-relevant antibody, BJ-8C, 24 hours prior to i.n infection with 3e5 CID_50_ PR8 S-Lux. As shown in **Figures 3a, b**, mice treated prophylactically with T1-3B had ~2-orders of magnitude lower bioluminescence in the airways compared with mice receiving BJ-8C (p<0.001; ANOVA with Dunnett’s post-test) or those that had not received any antibody pre-treatment. Importantly, the findings with PR8 S-Lux were mirrored with PR8 influenza, where an identical dose of T1-3B given prophylactically, gave complete protection against a lethal challenge (10,000 TCID_50_ (median tissue culture infectious dose); 1,000 LD_50_ (median lethal dose)) (**Figure 3c**). Together, these results show that bioluminescence analysis following PR8 S-Lux infection can be used to predict the prophylactic efficacy of neutralising antibodies *in vivo* without the restrictions imposed on wild-type influenza studies, that commonly need to be terminated to avoid undue suffering to the animals in the study.

### Validation of H5 and H7 S-Lux *in vivo* using known neutralising antibodies

**Figure 4:**
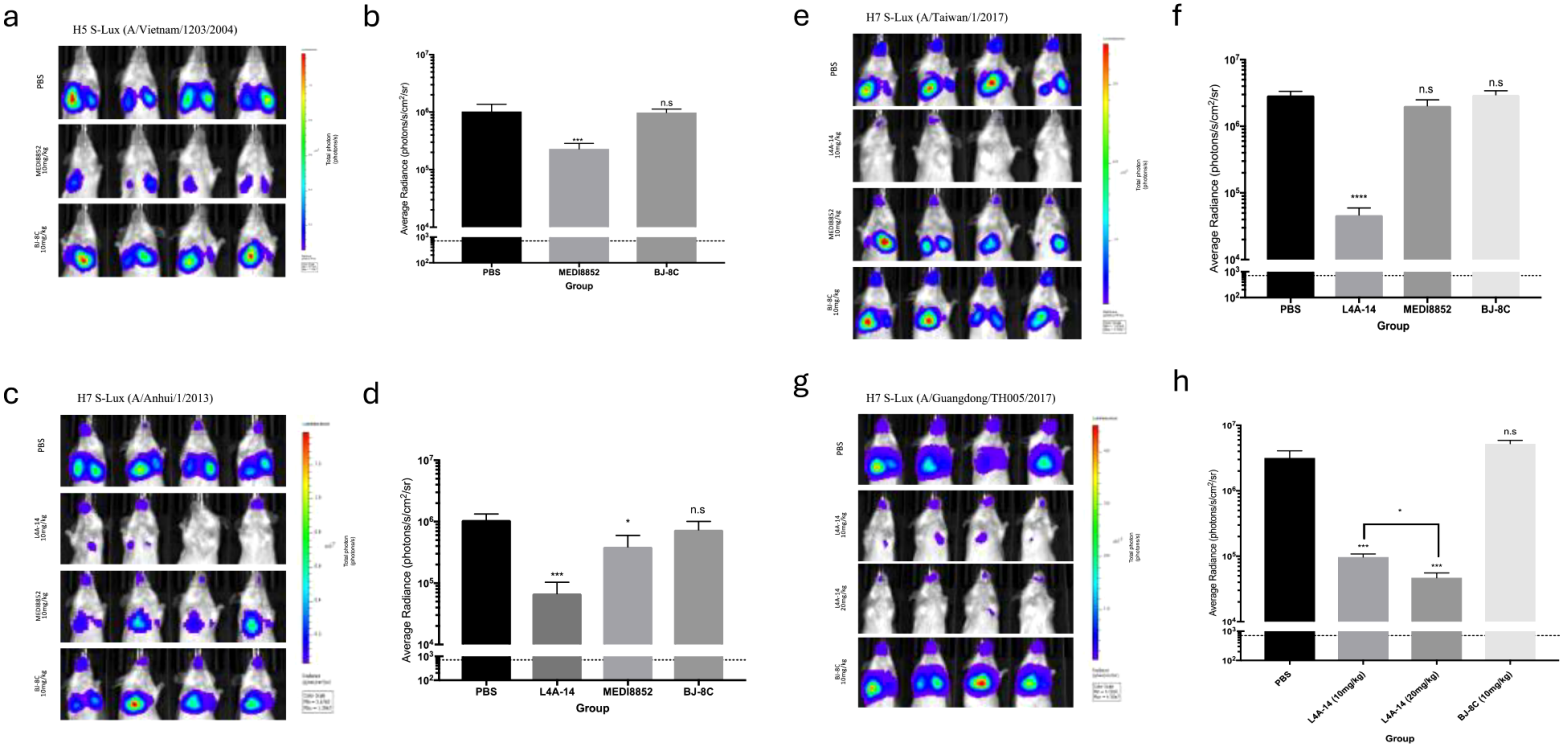
Validation of H5 and H7 S-Lux *in vivo* using known neutralising antibodies. Mice (n=4) were administered i.p. various antibodies or PBS, 24 h prior to i.n. infection with S-Lux pseudotyped with various H5 or H7 strains as indicated, followed by bioluminescence imaging of *flux* activity 24 h later. Data are presented as average radiance (photons/s/cm^2^/sr); each data point represents mean ± s.e.m (n=4). **(a, b)** show mice administered with 10 mg/kg of MEDI8852, non-relevant antibody BJ-8C, or PBS at 24 h prior to infection with 1e6 CID_50_ of H5 S-Lux (A/Vietnam/1203/2004), and imaged 24 h post-infection. **(c, d)** show mice administered with 10 mg/kg of MEDI8852, known neutralising antibody L4A-14, non-relevant antibody (BJ-8C), or PBS 24 h prior to infection with 1e6 CID_50_ of H7 S-Lux (A/Anhui/1/2013), and imaged 24 h post-infection. **(e, f)** show mice administered with 10 mg/kg of known neutralising antibody L4A-14, non-relevant antibody BJ-8C or PBS at 24 h prior to infection with 1e6 CID_50_ of H7 S-Lux (A/Taiwan/1/2017), and imaged 24 h post-infection. **(g, h)** show mice administered with 10 mg/kg or 20 mg/kg of known neutralising antibody (L4A-14), a non-relevant antibody BJ-8C or PBS 24 h prior to infection with 5e5 CID_50_ of H7 S-Lux (A/Guangdong/TH005/2017), and imaged 24 h post-infection. *p<0.05, ***p<0.001, ****p<0.0001. ns, not significant.

We subsequently evaluated the *in vivo* susceptibility of the panel of H5 and H7 S-Lux vectors to neutralising antibodies. To investigate H5 S-Lux, mice first received 10 mg/kg i.p dose of either the known pan-HA neutralising mAb MEDI8852 (21), an irrelevant BJ-8C mAb, or PBS as a control. Subsequently, mice were infected with 1e6 CID_50_ i.n of H5 A/Vietnam/1203/2004 S-Lux. At 24 hours post-infection, mouse respiratory bioluminescence was significantly lower in the group treated with MEDI8852 than when treated with BJ-8C or PBS (**Figures 4a, b**; p<0.0001, ANOVA with Dunnett’s post-test).

Similarly, mouse respiratory infection with H7 A/Anhui/1/2013 and H7 A/Taiwan/1/2017 S-Lux configurations was evaluated with 10 mg/kg i.p of MEDI8852, a novel H7 cross-reactive mAb L4A-14 (22), the irrelevant mAb BJ-8C or PBS. In both instances, S-Lux infection was efficiently inhibited with L4A-14 (**Figures 4c–f**; p<0.0001, ANOVA with Dunnett’s post-test). Interestingly, MEDI8852 had only a modest neutralising ability against H7 A/Anhui/1/2013 S-Lux (**Figures 4c, d**, p<0.05, ANOVA with Dunnett’s post-test) and no significant neutralising activity against H7 A/Taiwan/1/2017 S-Lux (**Figures 4e, f**, not significant, ANOVA with Dunnett’s post-test). For H7 A/Guangdong/TH005/2017 S-Lux, we evaluated two dosing levels with L4A-14 and showed that while both 10 mg/kg and 20 mg/kg significantly reduced H7 A/Guangdong/TH005/2017 S-Lux infection, (**Figures 4g, h**, p<0.0001, ANOVA with Dunnett’s post-test) the higher dose was superior (**Figures 4g, h**, p<0.0001, ANOVA with Dunnett’s post-test). Importantly, 20 mg/kg of L4A-14 has also been previously shown to protect mice against weight loss and conferred full protection against ~100 LD_50_ of the H7N9 (A/Guangdong/TH005/2017) influenza (22). Together, these results confirmed that H5 and H7 S-Lux can be used as surrogates for the respective wild-type influenza equivalents to aid prediction of prophylactic outcome of treatment with neutralising mAb *in vivo*.

### Use of S-Lux *to* study clearance of infected lung epithelia in immunised mice

**Figure 5:**
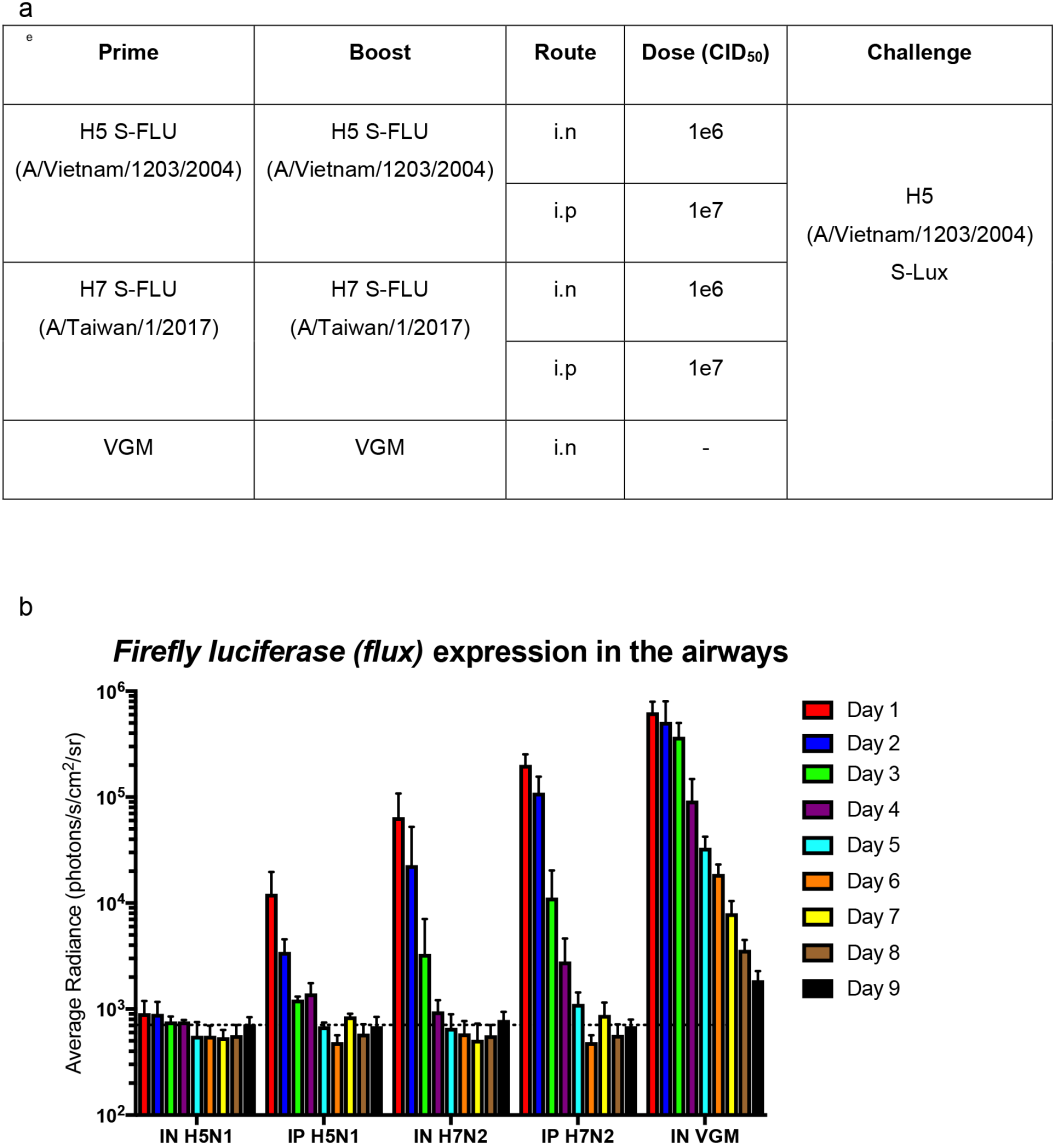
Validation of S-Lux to study lung clearance *in vivo* post S-FLU immunisation. **(a)** Table summarising the dosing schedule for the experiment. Mice (n=4-7) were immunised with two doses, two weeks apart, of H5 (A/Vietnam/1203/2004) S-FLU (matched) or H7 (A/Taiwan/1/2017) S-FLU, via either the intranasal (i.n) or intraperitoneal (i.p) route. A control group was included in which the mice were administered i.n with VGM (viral growth media). All mice were then challenged i.n with H5 (A/Vietnam/1203/2004) S-Lux 3 weeks post-immunisation. **(b)** Mice were imaged daily for up to 9 days post S-Lux infection with data presented as average radiance (photons/s/cm^2^/sr), mean ± s.e.m (n=4-7).

We further evaluated the use of S-Lux to study and monitor clearance of infected lung epithelia mediated by CTL (Cytotoxic T lymphocytes) response following immunisation. To investigate that, a previously published live attenuated influenza vaccine which was known to induce strong protective heterotypic T-cell responses, S-FLU (15) was used. Mice were primed and boosted via i.n (1e6 CID_50_) or i.p (1e7 CID_50_) dosing with H5N1 S-FLU (H5 (A/Vietnam/1203/2004); N1 (PR8) or H7N2 S-FLU (H7 (A/Taiwan/1/201; N2 (A/Victoria/316/2011)), 2 weeks apart (**Figure 5a**). At 2 weeks post-boosting, all mice received i.n challenge with H5 (A/Vietnam/1203/2004) S-Lux (1e6 CID_50_) and daily *flux* expression in the airways was measured. The results show that in both i.n and i.p routes where the mice were immunised with the “matched” S-FLU (H5N1) as the S-Lux, mice receiving i.n immunisation showed no expression of *flux* in the airways whereas mice receiving i.p immunisation showed *flux* expression at a log higher at 24h compared to i.n immunisation (**Figure 5b**). The results show that i.n immunisation of “matched” S-FLU was more effective in preventing influenza infection than i.p immunisation despite having lower serum neutralising antibody against H5 prior to challenge (EC_50_; i.n: <40; i.p: 540, **Supplementary Figure 2**).

In mice immunised with “unmatched” S-FLU (H7N2), both routes of administration showed no detectable serum neutralising antibody against H5 (**Supplementary Figure 2**) and did not block H5 (A/Vietnam/1203/2004) S-Lux infection in the airways. However, on day 1, both groups showed lower *flux* expression in the airways compared with the VGM (viral growth media) control group, suggesting that clearance of infected cells had happened before the first measurement (day 1). In general, both groups have more rapid clearance of *flux* signal compared with VGM control mice. Intranasal immunisation seems to exert a stronger (reduction by half a log on day 1) and more rapid clearance of signal in the lung (by a day) compared with i.p immunisation.

### Use of S-Lux pseudotyped with Ebola virus glycoprotein *in vivo*

**Figure 6:**
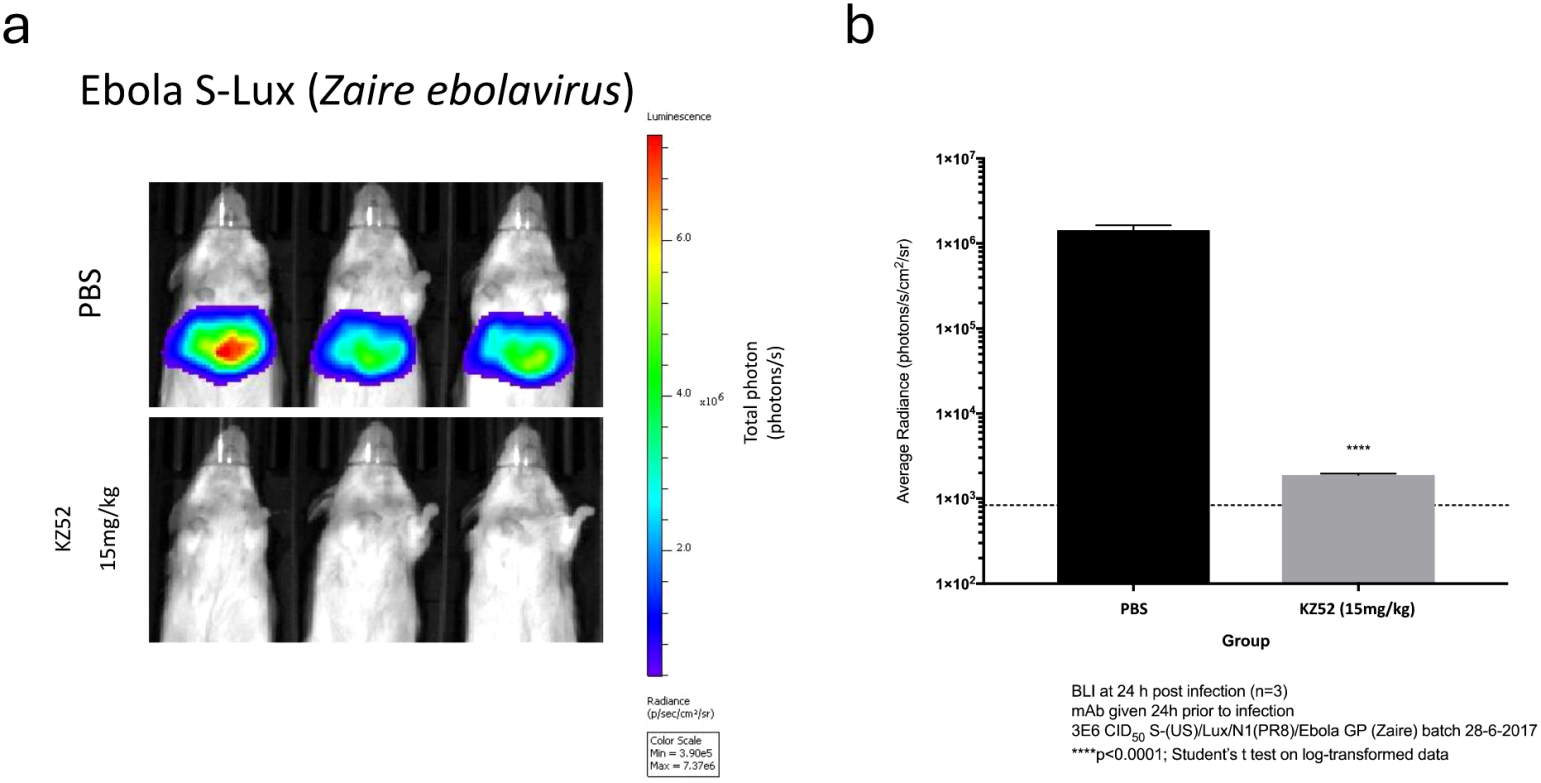
Validation of Ebola S-Lux (ES-Lux) *in vivo* using known neutralising antibody. Mice (n=3) were administered i.p with 15 mg/kg of known neutralising antibody KZ52 or PBS 24 h prior to i.v infection with 3.6e5 CID_50_ of ES-Lux and **(a)** imaged 24 h post-infection, with **(b)** data presented as average radiance (photons/s/cm^2^/sr), mean ± s.e.m (n=3). ****p<0.0001.

Following the successful use of S-Lux with influenza pseudotypes, we tested if glycoproteins from other infectious diseases could also be used to pseudotype S-Lux generating a reagent to facilitate the evaluation of anti-viral therapies targeted at the cognate glycoprotein and/or glycoprotein/receptor complex. As a pertinent exemplar, we investigated the utility of the S-Lux system with the Ebola virus (EBOV) glycoprotein. Ebola virus is a highly virulent and lethal human pathogen, which caused 11,316 deaths out of the 28,639 documented cases (~40% fatality) during the 2014–2016 Ebola outbreak in West Africa (23). Importantly, EBOV pseudotyped influenza has been shown to use the same entry pathway as the wild type Ebola virus (17), and can be useful as a surrogate to study EBOV entry. To facilitate the study of prophylaxis for EBOV in accessible low containment laboratories, we pseudotyped S-Lux with the glycoprotein from the *Zaire* EBOV (Makona variant). To evaluate *Zaire* EBOV S-Lux (ES-Lux), we dosed mice with 15 mg/kg of the EBOV neutralising mAb KZ52, which is perhaps one of the most extensively studied anti-Ebola antibody (24), or with PBS as a mock control 24 h prior to intravenous (i.v) infection with ~3.6e5 CID_50_ of the ES-Lux. The result showed that at 24 h post-infection, mice that received PBS displayed high bioluminescence signal, which as expected was localised to the liver, whereas mice receiving the KZ52 mAb showed approximately three orders of magnitude lower bioluminescence, which was just slightly above background signal (**Figure 6**; P<0.0001, t-test). This demonstrates that *Zaire* ES-Lux, similar to EBOV (25), has a high tropism for the liver after i.v. delivery. In addition, the results show that the relevant antibody against Ebola GP is also capable of blocking infection of *Zaire* ES-Lux when given prophylactically.

## Discussion

The principal aim of this study was to develop a strategy to facilitate evaluation of treatments for lethal human pathogens, without recourse to high biosafety containment facilities. Studies utilising non-invasive bioluminescence imaging of live animals can be performed in standard, readily available, BSL-2 facilities and could perhaps serve as a preliminary screen prior to expensive wild-type infection challenge studies. We developed a surrogate recombinant influenza virus capable of expressing luciferase so that non-invasive *in vivo* imaging can be used as a marker of infection, for testing of neutralising antibodies and potentially other molecules against pandemic influenza viruses. Although reporter-expressing influenza A viruses have been described previously (9–12), to our knowledge, our work demonstrates the first non-replicating avian H5 and H7 influenza surrogates capable of expressing *flux*, which are also sufficiently safe for use in low containment facilities. Using our protocol, S-Lux pseudotyped with HA from different strains of influenza, especially those that possess pandemic risk, can be readily produced by supplying the selected HA *in trans* during production. The simplicity of this system means that it could expedite the testing of prophylaxis when new influenza strains arise in humans.

Proof-of-principle experiments showed that a prototype H1N1 S-Lux (PR8 S-Lux) infects cells *in vitro* to express *flux* (**Figure 1c**) and is able to infect the mouse airways, displaying high levels of *flux* as early as 24 h post-infection in the lung (**Figure 2**), which is considerably earlier than weight loss can be detected in conventional influenza virus challenge studies (**Figure 3**). Using a mouse model of infection, we showed that pre-treatment with a known neutralising mAb (T1-3B at 10 mg/kg), significantly reduced *flux* expression in the airways (**Figures 3a, b**). Importantly, the selected T1-3B dose was able to protect mice against lethal challenge (~1,000 LD_50_) of A/PR/8 influenza (**Figures 3c, d**). The correlation between results from *in vivo* imaging of S-Lux and lethal challenge with influenza confirms that this model can be used to predict potential for prophylactic efficacy of neutralising mAbs, without provoking profound illness in the infected mice.

We then tested H5 and H7 pseudotyped S-Lux using the published pan-HA stem targeting mAb MEDI8852, or a novel H7 mAb isolated from a recovered patient, L4A-14 that targets the H7 Receptor Binding Site (22). The MEDI8852 mAb has been shown to cross-react against all HA subtypes (21, 26). Here, we show that administration of MEDI8852 resulted in a statistically significant reduction in *flux* activity in the murine airways infected with H5 S-Lux (A/Vietnam/1203/2004) (**Figures 4a, b**) and H7 S-Lux (A/Anhui/1/2013) (**Figures 4c, d**), whereas administration of BJ-8C did not block activity. Using our system, the neutralisation of MEDI8852 against H7 (A/Anhui/1/2013) was less pronounced than H5 (A/Vietnam/1203/2004) (**Figures 4a–d**) which is consistent with previously published *in vitro* neutralisation data using equivalent influenza viruses (21, 27). Surprisingly, MEDI8852 did not block *flux* activity from H7 S-Lux (A/Taiwan/1/2017) (**Figures 4e, f**). The reason for this is unknown, although MEDI8852 is known to have a weaker neutralising efficacy against H7 viruses in general (21). It is also possible that MEDI8852 has reduced neutralising efficacy against the more recent H7 isolate (A/Taiwan/1/2017). This requires further investigation as, here, a somewhat higher dose of H7 S-Lux (A/Taiwan/1/2017) was used in the experiment reported here (1e6 CID_50_), approximately 10 to 100-fold higher than doses of wild type H7 typically used in challenge studies (28, 29). Interestingly, in mice treated with relevant mAbs, the decrease in bioluminescence observed in the nasopharyngeal region was not as pronounced as that observed in the lungs, possibly due to the poor distribution of IgG to the upper airways as reported previously by others (10).

We also showed that the novel mAb L4A-14 significantly reduced *flux* activity with all H7 S-Lux configurations evaluated (**Figures 4c–h**) and under the conditions used, its neutralising effect appears greater than MEDI8852 (**Figure 4d**). This is perhaps not surprising as it has been previously observed that HA head-targeting mAbs (such as L4A-14) typically exhibit 10 to 100-fold greater neutralising effects than stem-targeting mAbs such as MEDI8852 (18). It is also worth noting that L4A-14, which significantly reduced *flux* activity of H7 S-Lux (A/Guangdong/TH005/2017) at both 10 mg/kg and 20 mg/kg, has been shown to provide 80% and 100% protection in mice against ~100LD_50_ of the wild type H7N9 equivalent (22). This further supports the model in which *flux* expression mediated by S-Lux can be used to predict prophylactic efficacy of neutralising mAbs.

One interesting observation in this study was the difference in airways tropism observed with different HAs. All three H7 S-Lux viruses infected both the upper and lower airways of the mice (**Figure 4**), whereas H5 S-Lux infected only the lower airways when the same dose of virus was given, suggesting that H5 has little or no tropism for the upper airways of mice. These observations also suggest that our model could potentially be used to study tropism of influenza viruses, especially of newly isolated strains or those that possess pandemic risks. This could be further confirmed in the ferret model which is known to have sialic-acid distribution in the airways similar to humans to better predict the airways tropism of novel influenza strains in humans (30).

After showing S-Lux can be used to study neutralising antibodies against influenza *in vivo*, we investigated if S-Lux can be used to study clearance of infected cells (presumed to be mediated by CTL in immunised mice). We utilised S-FLU which has been previously shown to induce strong protective heterotypic T-cell responses against multiple influenza strains (15, 19, 20). Mice immunised twice i.p with 10^7^ TCID_50_ “matched” S-FLU (H5N1) were expected to possess neutralising antibody against H5 (A/Vietnam/1203/2004) S-Lux. By contrast, mice immunised with a 10-fold lower dose (10^6^ TCID_50_) i.n. develop a strong local CTL response but generate a weak or undetectable systemic neutralising antibody response (15). Despite high serum neutralising titre in the i.p group compared to i.n administration (EC_50_; i.n: <40; i.p: 537, **Supplementary Figure 2**), our results indicated that low dose i.n immunisation elicits a stronger infection blocking and clearing effect against homologous H5 (A/Vietnam/1203/2004) S-Lux than high dose i.p immunisation (**Figure 5**). This result highlights the importance of the route of influenza vaccine administration and local immunity. Although not tested in this study, we speculated that i.n immunisation of S-FLU elicits strong local antibody response, probably of IgA subclass, which completely prevented S-Lux infection. Our results also suggested that serum neutralising titre might not accurately predict the depth of homotypic protection against influenza infection in the lungs.

In both the groups in which the mice received “unmatched” heterotypic S-FLU (H7N2) to the challenge H5 (A/Vietnam/1203/2004) S-Lux, no serum neutralising antibody against H5 was detected prior to challenge (**Supplementary Figure 2**), supported by high initial expression of *flux* in the airways following H5 (A/Vietnam/1203/2004) S-Lux challenge (**Figure 5**). Both groups showed a more rapid clearance of *flux* signal in the airways compared to mock vaccinated (VGM) group, a result which mirrors the “partial heterotypic immunity” between influenza A viruses first described by Schulman and Kilbourne in 1965 (31). A new finding that has not been shown with S-FLU vaccine was that the response (presumed to be Cytotoxic T-cells) induced via the systemic route (i.p) at high dose (10^7^ TCID_50_) is also capable of improved clearance of infected lung epithelia, albeit at a slower rate than a 10x lower dose (10^6^ TCID_50_) given locally (i.n). Collectively, our results show that S-Lux can be used to study T cell-based influenza vaccines.

To extend the utility of our system to other infectious diseases, we evaluated the S-Lux system for Ebola. Using the neutralising mAb KZ52, which has shown protection in animal models against EBOV challenge (24), we observed blocking of infection by ES-Lux (**Figure 5**) (24, 32), indicating that ES-Lux is a useful preliminary tool to evaluate prophylactic efficacy of neutralising mAb and potentially drugs targeting wild type EBOV infection. We note that the *flux* activity in mice following ES-Lux delivery is localised to the liver (**Figure 5**), a finding consistent with previous reports using the Ebola virus-like particle reporter system (33), and which also correlates with the natural tropism of wild type EBOV. High viral load was detected in the livers of mice infected with wild type Ebola virus (34) and extensive pathological changes were found in livers from non-human primates and humans infected with Ebola virus (reviewed in (35, 36). Compared with the published Ebola virus like particle reporter system (33) where use is exclusive to testing EBOV, the ES-Lux system has increased versatility to allow pseudotyping of other enveloped viruses requiring BSL-4 facilities, including Lassa virus, Marburg virus and Nipah virus, thereby facilitating testing of prophylaxis in low containment facilities.

One limitation of our approach is the non-replicating nature of S-Lux and ES-Lux, which means that it is not possible to study viral replication or therapeutic efficacy directly. There is a possibility that the inhibition of S-FLU and ES-FLU infection may overestimate the degree of neutralisation compared to wild-type virus owing to the increased sensitivity of pseudotyped viruses to antibody-mediated blockade. This point should be acknowledged. However, if such a difference exists, it is an inherent and unavoidable limitation of this safe model. Nevertheless, S-Lux constitutes a useful tool to study neutralising mAbs and anti-viral drugs that block entry of influenza and Ebola virus. For testing prophylaxis against standard influenza strains that can be handled in low containment facilities such as PR8, our system can shorten the duration of the overall study by using bioluminescence imaging at 24 h post-infection instead of monitoring weight loss over 2 weeks. This approach also reduces the total number of animals required, and the severity of illness induced, both important considerations for the use of animals in such studies. Importantly, our system offers the opportunity to test neutralising mAbs against potential pandemic HAs such as H5 and H7 and other highly infectious pathogens such as EBOV in commonly available BSL-1 or 2 facilities, removing the constraint of limited access to high containment testing facilities.

In conclusion, our approach serves as a simple, robust, time-responsive and cost-effective tool to screen for prophylaxis against pandemic influenza in low containment facilities, which could greatly facilitate vaccine or prophylactic drug or antibody development against pandemic influenza and EBOV.

## Methods

### Generation of S-Lux

S-Lux was produced based on the described method (15, 17) with modifications highlighted in **Figures 1a, b**. The codon optimised *flux* sequence [GenBank accession no. CAA59282.1] was synthesised between appropriate *NotI* and *EcoRI* restriction sites and ligated into the S-FLU expression cassette. In brief, recombinant S-Lux viruses on the A/PR/8/1934 background were produced by transfection of HEK 293T cells as described (15, 37) with 1µg of each of the following plasmids pPol plasmids encoding the vRNA segments (pPol_PB1-PR8, pPol_PB2-PR8, pPol_PA-PR8, pPol_NP-PR8, pPol_NS-PR8, pPol_M-PR8, pPol_ vRNA), 4 core initiators to supply essential viral proteins in trans for vRNA replication and viral packaging (pCDNA3.1_PB1-PR8, pCDNA3.1_PB2-PR8, pCDNA3.1_PA-PR8, pCDNA3.1_NP-PR8), pCDNA3.1_H1_HA-PR8 (to supply HA in *trans* on the producer HEK293T cells) and pPol/S-US-HA-flux), and cloned twice by limiting dilution in MDCK-SIAT1 cells, transduced to express coating haemagglutinin from PR8 (Cambridge Strain) [GenBank accession no. CAA24272.1] to provide the pseudotyping haemagglutinin *in trans*. Viruses were then propagated in appropriate transduced MDCK-SIAT1 cells or MDCK-E-SIAT1 coated in H5 from A/Vietnam/1203/2004 [GenBank accession no. EF541403.1, (20)], H7 (A/Taiwan/1/2017 GISAID EPI917065; A/Anhui/1/2013 GISAID EPI439507; A/Guangdong/TH005/2017 GISAID EPI926825) or Ebola GP (Zaire ebolavirus Makona wt/GIN/2014/KissidougouC-15) [GenBank accession no. KJ660346.1; (17)], respectively, to generate S-Lux or ES-Lux. All HA sequences originally containing a polybasic cleavage site were converted to dependence on trypsin; for H5: PQRETR/GLFGAIA and H7: PEIPKGR/GLFGAIA (See OFFLU at http://www.offlu.net/: Influenza A Cleavage Sites, 31st Jan 2018).

### Production of recombinant human monoclonal antibodies

Recombinant human IgG used in this study was produced by transient transfection of the IgG heavy and light chain expression plasmids (AbVec) in HEK293T grown in suspension in serum free media. Several of the IgG (BJ-8C and L4A-14) were produced using the ExpiCHO expression system (Thermo Fisher Scientific) according to the manufacturer’s protocol. IgG was then purified using the HiTrap MabSelect SuRe column (GE Healthcare) according to the manufacturer’s protocol. The VH of the MEDI8852 used in our study differs from the original sequence by a single amino acid (PKLLIYA to PKLLLYA) due to a misprint in the figure in (21).

### Titration of S-Lux

S-Lux and ES-Lux were titrated as CID_50_ as previously described (17) with slight modifications. Briefly, harvested supernatants containing S-Lux were titrated in a 2-fold serial dilution in viral growth media (VGM; DMEM-penicillin-streptomycin-0.1% BSA without trypsin) across a black flat-bottom 96-well plate seeded with 3e4 MDCK-SIAT1 cells. The plate was incubated at 37°C overnight and imaged for bioluminescence as described below. The dilution of virus giving 50% of the maximum plateau bioluminescence signal (EC_50_) was calculated by linear interpolation. The CID_50_/ml was calculated from the EC_50_ dilution and the number of cells seeded per well (3e4 cells).

### Microneutralisation assays

Microneutralisation assays were performed according to (15). Briefly, influenza viruses were diluted in VGM and titrated to give plateau expression of *firefly luciferase* in 3e4 MDCK-SIAT1 cells after overnight infection in 96-well flat-bottomed plates. Serial (2-fold) dilutions of mAb were incubated with S-Lux for 2 h at 37°C. A total of 3e4 MDCK-SIAT1 cells were then added in 100 µl of VGM and incubated overnight at 37°C and *flux* expression determined (as below). Titres were reported as fold-dilution of antibody that resulted in 50% reduction in *flux* expression (EC_50_).

### Animal experiments

Animals used in this study were purchased from Envigo Ltd. (Shaw Farm, Bicester, United Kingdom) and procedures carried out at the Biomedical Services Unit (BMS) (University of Oxford, United Kingdom) under the terms of the Animal (Scientific Procedures) Act 1986. Mice were housed in accordance with the UK Home Office ethical and welfare guidelines and fed on standard chow and water *ad libitum*. Female BALB/c mice were used at 4 to 6 weeks of age. Antibody or vehicle (PBS) was delivered via intraperitoneal (i.p) delivery in a total volume of 500 µl. For delivery of S-Lux, mice were anaesthetised with isoflurane and 50 or 100 µl of S-Lux was delivered via the intranasal (i.n) route. For delivery of ES-Lux, mice were restrained and intravenous (i.v) delivery was performed via tail vein injection. For wild type influenza challenge study, mice were infected i.n with 10,000 TCID_50_ of wild type A/PR/8/1934 (Cambridge) virus and a humane endpoint of weight loss and clinical score was used for mice that would otherwise have succumbed to infection. Animals were assessed for clinical score in terms of mobility, appearance, and breathing intensity. Mice reaching 20% weight loss from the pre-challenge body weight and/or a morbid clinical score were euthanised. Mice were euthanised by gradual fill of the chamber using compressed CO_2_ (displacement rate of 30-70% chamber volume per minute).

### Bioluminescence imaging

For imaging of cell culture plates, supernatant was removed from all wells and washed with PBS. A working stock of 1x D-Luciferin (1.5 mg/ml) diluted in 100 µl D-PBS was then added to each well and incubated for 5 min before images were captured. For live animal imaging, mice were anaesthetised with isoflurane before i.n delivery of 100 µl of 100x (15 mg/mL) D-Luciferin for S-Lux studies and i.p delivery of 300 µl of 100x (15 mg/mL) D-Luciferin for ES-Lux studies. Images were captured after 5 min incubation. During imaging, anaesthesia was maintained via nasal delivery of 2.5% isoflurane. All images were acquired using the Xenogen IVIS Lumina LT Series III using the automated settings and analysed with LivingImage 4.5 software package (XenonCorp). For mouse imaging, regions of interest (ROIs) were drawn by creating a circle region around the chest area. For cell culture plates, ROIs were drawn using the standard grids with matrices consistent with cell culture plates. Bioluminescence signal from ROIs were defined and expressed as average photon flux (photons/s/cm^2^/sr). Data are presented as mean ± SEM.

### Statistics

Column and time-course data are presented as mean ± SEM. Differences between treatment groups were determined on log-normalised data using the t-Test, ANOVA with *post hoc* test for linear trend, or ANOVA with Dunnett’s multiple comparison test as appropriate. P-values of less than 0.05 were deemed statistically significant. Analyses were performed using GraphPad Prism 10.

## Data Availability

The original contributions presented in the study are included in the article/**Supplementary Material**. Further inquiries can be directed to the corresponding author.
