# Supplementary material for: Single-cycle, pseudotyped reporter influenza virus to facilitate evaluation of treatment strategies for avian influenza, Ebola and other highly infectious diseases in vivo
Source: Front Immunol. 2025 Jul 10;16:1608074. doi: 10.3389/fimmu.2025.1608074 (PMC12286815; doi:10.3389/fimmu.2025.1608074)
Supplement: Supplementary file 1 [file DataSheet1.pdf]

frontiers

Supplementary Material

Supplementary Figures

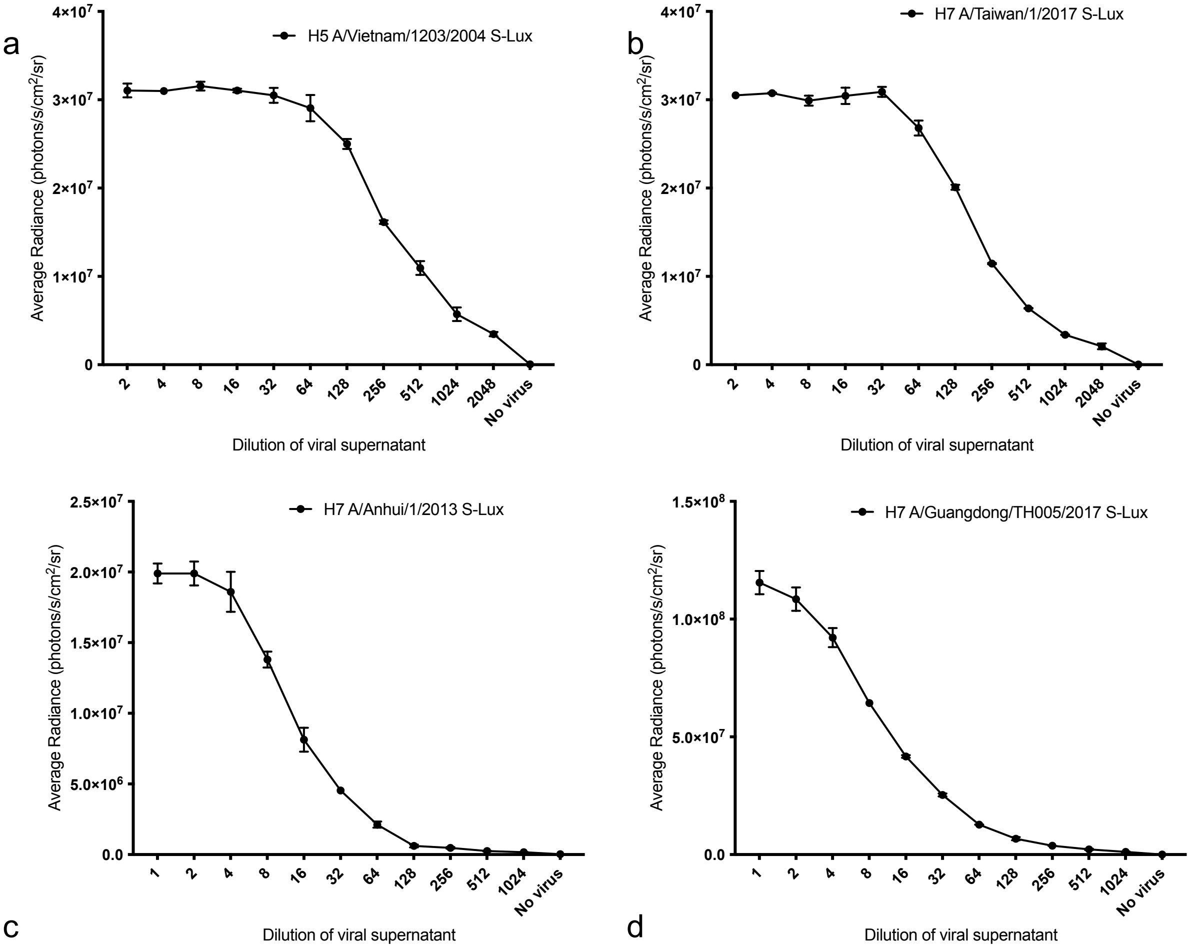

Supplementary Figure 1 Firefly luciferase expression of H5 and H7 S-Lux viruses *in vitro*. Monolayers of MDCK cells seeded in 96-well plates were infected with dilutions of (a) H5 S-Lux (A/Vietnam/1203/3004), (b) H7 S-Lux (A/Taiwan/1/2017), (c) H7 S-Lux (A/Anhui/1/2013) or H7 S-Lux (A/Guangdong/TH005/2017) and bioluminescence was measured 24 h post infection. Each data point represents the average of two readings.

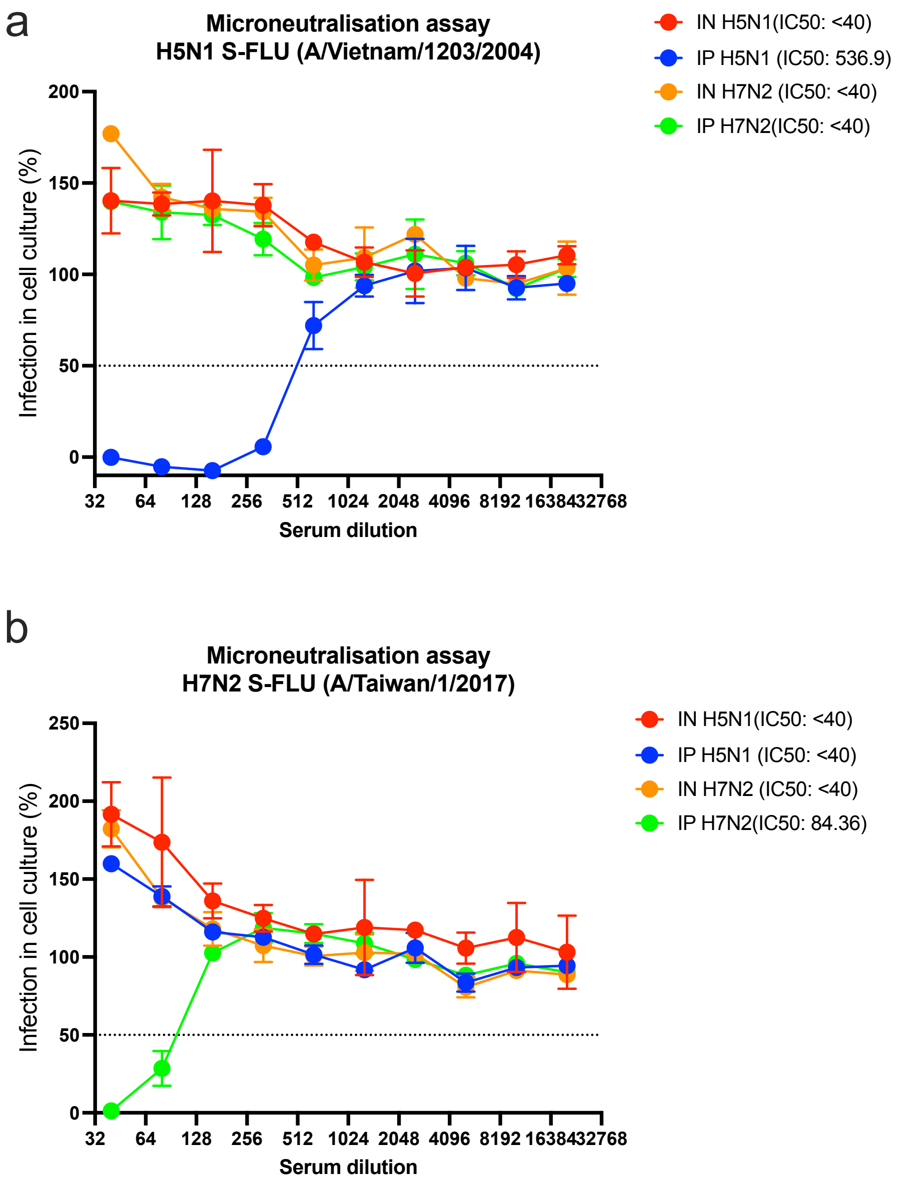

Supplementary Figure 2 Serum neutralisation activity of mice immunised with H5 and H7 S-FLU. Microneutralisation assays against H5N1 (a) or H7N2 (b) of the sera of mice immunised with S-FLU (as indicated in Figure 5a). Each data point represents the average of two readings.
